# Restoring sight: how cataract surgery improves the lives of older adults

**Published:** 2008-06

**Authors:** Sarah Polack

**Affiliations:** Research Fellow, International Centre for Eye Health, London School of Hygiene and Tropical Medicine, Keppel Street, London WC1 7HT, UK. Email: sarah.polack@Lshtm.ac.uk

**Figure:**
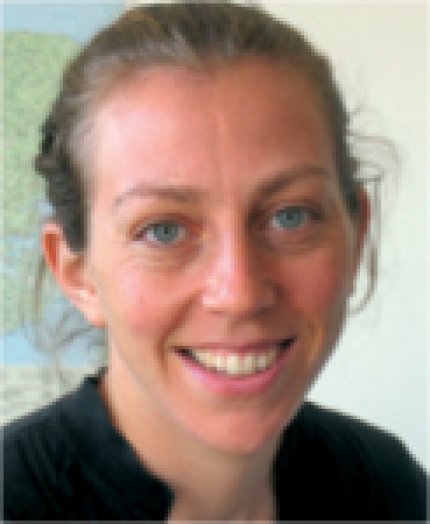


The fundamental aim of most ophthalmic interventions in later life is to improve the quality of patients' lives, whether through sight-restoring cataract surgery or the provision of visual aids. Amidst the pressures of targets, outputs, and backlogs, this may be all too easily forgotten. It is therefore important to step back and remember just how important good vision is in the lives of older adults.

Vision loss has a major negative impact on the quality of older people's lives. Sight remains as valued and important in later life as at any other age and its loss is one of the things older people fear most. Improving access to eye care services for this age group, as well as older people's uptake of such services, is therefore very important.

This article takes a closer look at some of the ways in which vision loss and blindness can affect the lives of older adults; it also highlights the positive impact sight-restoring cataract surgery has on older people's lives.

## The impact of vision loss

It is usual to describe vision loss in clinical terms (such as visual acuity, for example) or in epidemiological terms (such as the prevalence of severe visual impairment or blindness in a population). But what does low vision or blindness really mean for an older person?

In order to find out, in-depth interviews were conducted in Kenya and Bangladesh with adults over the age of 50 who had recently undergone cataract surgery. This formed part of a wider study by the International Centre for Eye Health on the impact of cataract surgery on quality of life and poverty. Some of the findings are presented in this article.

These findings support previous research using patient-reported outcome measures (PROMs) in both high- and low-income settings.^1^^,^^2^ PROMs are questionnaires which aim to discover the impact of health conditions or treatment as perceived by the patient, rather than from clinical measures alone.

### Difficulties with daily activities

Our research showed, unsurprisingly, that older adults with visual impairment had greater difficulties with their daily activities than those without impairment. These difficulties varied considerably according to older adults' lifestyle, environment, and social support, as well as the severity of their vision loss.

“Before the operation I felt as if I was put into a jail that I couldn't escape. Many times I would take tea and burn myself as I couldn't see the handle. It was horrible.” (Kenya)

“[Before surgery] I never used to go to meetings, bazaars, and weddings. I could not light fire for myself and I never used to know the difference between crops and weeds. I couldn't even bathe myself. I could not differentiate money given to me and used to be given less change often because I couldn't see.” (Kenya)

### Reduced earning capacity

Older people with visual impairment are less able to earn a living or contribute to the household. These restrictions have obvious economic implications. Amongst the people we interviewed in Bangladesh, a 70-year-old man told us that because of his poor eyesight (from cataract) he was rejected as a day labourer and lost his job as an imam. A 58-year-old woman from the same district reported that she used to make handicrafts to sell, but because of her eyesight “they were no longer of good quality.”

### Limited participation and social isolation

Older people with visual impairment are less likely to engage in social and family life. Participation in activities, relating to both work and leisure, is well established as an important factor in the wellbeing and happiness of older adults.^3^ Restrictions on participation not only have economic implications, but they can also impact on many social and psychological aspects of a person's life. Reduced opportunities for interaction and involvement with social networks, for example, could lead to feelings of isolation and lack of social support.

“Before [surgery] my friends didn't often come to visit; they were not interested because they knew I couldn't see.” (Kenya)

“I was gradually transforming into a neglected person by my family members.” (Bangladesh)

“…when I used to ask for help they used to get annoyed and tell me to do it on my own. I used to sit helpless in the corner because I could not see.” (Bangladesh)

To further compound this impact, there tends to be a disproportionately large number of older people among the more neglected and marginalised members of society. It is likely that visual impairment exaggerates this situation.

### Anxiety and depression

There is some indication of a relationship between age-related vision loss and depression in later life.^4^ For example, older adults in Kenya with visual impairment due to cataract were three times more likely to report anxiety and depression compared to people of the same age with normal vision.^5^

“I couldn't do anything. Even if I wanted to go to the road, someone had to hold my hand and take me. I didn't appreciate anything and felt really sad.” (Kenya)

In addition, research shows that restricted capacity to contribute to the household or community can lead to feelings of loss of independence, feelings of being a burden on others, and reduced social status and self-esteem.^6^

“I was finished without my sight as I was the breadwinner and I was not able to give my family anything. I felt embarrassed about my eyes.” (Kenya)

### The impact on others

The significant contribution of older people to the wellbeing of others and to wider society is well documented, but it is under valued and all too often forgotten.

In low-income countries there is evidence that, in poor households, all members - regardless of age - contribute to the basic needs of the household.^8^ Taking care of children is just one example of how older people contribute: the benefits of this are likely to be multiple and include enabling a parent to continue in paid employment. In countries heavily affected by HIV and AIDS, older people often have to care both for their sick children and for their grandchildren.

When older adults become visually impaired or blind, their ability to contribute economically, and to social and family life, is greatly reduced (as some of the above quotes show); this loss can be felt quite keenly by other members of the household.

## The positive impact of eye surgery

There is a misconception (seemingly also present among older people themselves) that sight is less important in older age. Some also believe that vision loss and its restrictive impact should be expected - and accepted - as part of the ageing process. This is clearly not the case. Research has shown that cataract surgery has a far-reaching impact on the quality of older people's lives.

**Figure:**
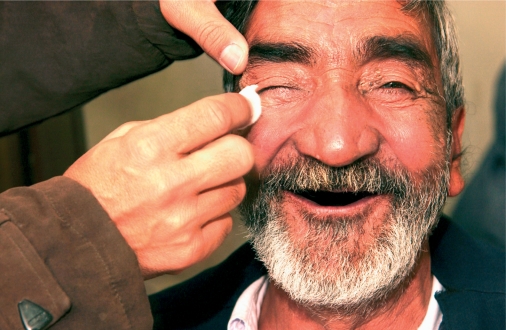
**Cataract surgery has restored this man's vision. PAKISTAN**

Studies in various countries using PROMs have shown a marked improvement in the physical functioning and psychological well-being of older people.^9^ It has also been shown that cataract surgery continues to be beneficial even amongst the very old.^10^ In our study in Kenya and Bangladesh, the very old or those suffering from other health problems or diseases expressed that their improved vision was of huge value.

“I had not seen two of my children, who were born when I was blind. I couldn't read reports of my children from teachers at school. I could not raise money to educate my children. After surgery I felt like a small child born again. I really celebrated; so did my family. The first thing I wanted to see was my two grandchildren. It was amazing! They were young and very beautiful.” (Kenya)

The following are some of the other benefits that older people in our study experienced after receiving sight-restoring cataract surgery.

### Greater social inclusion and participation

Patients reported an increase in their participation in social activities.

“After surgery I was very happy because I could visit my friends and attend village meetings, bazaars, and functions like weddings.” (Bangladesh)

“I feel happy because even though I'm old, at least I can see people.” (Kenya)

This renewed capacity to participate in social events was considered a major beneft; it was also linked to feelings of happiness and confidence.

“Now I have a very good relationship with my family members and others. Before the operation I used to feel shy to socialise. Now I do not feel that.” (Bangladesh)

### Engagement in productive activities

Following cataract surgery, many interviewees were able to once again engage in productive activities, be that driving a taxi, working on their farmland, or contributing to the upkeep of the household.

“After surgery I completely got back my eyesight, but because of my old age I cannot do heavy work. But I can help with the household chores so my family can concentrate on earning money.” (Bangladesh)

“I was able to do my business again making locks and knives to sell. This helped me to get money for my family.” (Kenya)

### Increased self-esteem

In addition to, and perhaps as a result of, increased participation in social and work activities, a new level of independence was commonly reported by older people after surgery. Associated with this was a feeling of increased self-esteem and respect from others.

“Earlier [before surgery] I used to feel helpless, but I am not feeling like that anymore. I am confident now. I can earn my living by housekeeping.” (Bangladesh)

“I do not need to depend on anyone. I can do my own work. Besides, people used to neglect me earlier, but now they respect me very much.” (Bangladesh)

### Improved communication and relationships

Other, perhaps less obvious, benefits from cataract surgery included improved communication with others, stronger social relationships, and being more trusting of others:

“Now when I meet someone in the community, I can say hello and genuinely feel I have communicated and I can tell whether people are truly saying hello with a good heart. Before [surgery] I didn't want to talk to people because I didn't know whether to trust if they were being true. It's easier to talk to people now.” (Kenya)

“My family is more harmonised and able to work together better. We can discuss issues better now as I can see them [his family] clearly.” (Kenya)

### Wider impact of surgery on other members of the community

It should also be remembered that the benefits of sight-restoring cataract surgery extend beyond the individual to the wider household; surgery has positive social and financial consequences. Restoring one person's sight may free up time and alleviate the anxiety of others, for example those who previously needed to remain at home to look after or assist an older blind relative.

“We used to worry when we used to leave him alone at home. Now it is not happening. We can go to the workplace without worrying. Besides, he needed help with eating, travelling, sleeping, bathing … now it is not necessary. So we are very happy.” (Bangladesh)

“The family is happy because we are able to attend other duties now that she is independent. We are able to go about our duties without worry or hurrying back to the house to attend to her.” (Kenya)

### Conclusion

In the absence of appropriate intervention (whether spectacles, surgery, low vision care, or rehabilitation), visual impairment has a substantial negative impact on many factors that contribute to the quality of life of older adults. These range from perhaps the more obvious effects of a reduced capacity to undertake day-to-day activities, through to a more subtle influence over psychological wellbeing.

The significant physical and psychological benefits of cataract surgery, in terms of the quality of a person's life, should serve as a powerful reminder of the value of ophthalmic interventions for the older adult.
